# Natural, longitudinal recovery of adults with COVID-19 using standardized rehabilitation measures

**DOI:** 10.3389/fnagi.2022.958744

**Published:** 2022-08-25

**Authors:** Carrie A. Ciro, Shirley A. James, Hillary McGuire, Vince Lepak, Susan Dresser, Amy Costner-Lark, Wanda Robinson, Terrie Fritz

**Affiliations:** ^1^Department of Rehabilitation Sciences, College of Allied Health, The University of Oklahoma Health Sciences Center, Oklahoma City, OK, United States; ^2^Department of Rehabilitation Sciences, The University of Oklahoma Health Sciences Center at the OU-Tulsa Schusterman Campus, Tulsa, OK, United States; ^3^College of Nursing, The University of Oklahoma Health Sciences Center, Oklahoma City, OK, United States; ^4^Anne and Henry Zarrow School of Social Work, Dodge Family College of Arts and Sciences, The University of Oklahoma, Norman, OK, United States

**Keywords:** COVID-19, rehabilitation, multi-disciplinary, function, cognition, mobility

## Abstract

**Background:**

While studies recommend rehabilitation following post-hospitalization recovery from COVID-19, few implement standardized tools to assess continued needs. The aim of this study was to identify post-hospitalization recommendations using an interdisciplinary needs assessment with standardized rehabilitation measures. A secondary aim was to use these tools to measure recovery over a 30-day period.

**Materials and methods:**

Using a 30-day longitudinal design, we completed weekly rapid needs assessments in this convenience sample of 20 people diagnosed with COVID-19 discharged from the hospital to home. We computed summary statistics and used the Wilcoxon Signed Rank Test to assess change over the 4-week course of the study with alpha level = 0.05.

**Results:**

Our sample (65% male, 47% over 50 years of age, 35% White, 37% with a confirmed diagnosis of diabetes, and 47% obese) included no patients who had required mechanical ventilation. Initial assessments demonstrated the majority of our participants were at an increased risk of falls, had disability in activities of daily living (ADL) and instrumental activities of daily living (IADL), mild cognitive impairment, and dyspnea. At the 30-day follow-up, most were independent in mobility and basic ADLs, with continued disability in IADLs and cognitive function.

**Discussion:**

In this sample of patients who were not mechanically-ventilated, early and individualized rehabilitation was necessary. The results of this study suggest patients would benefit from a multi-disciplinary team needs assessment after medical stabilization to minimize fall risk and disability, and to prevent secondary complications resulting from post-hospital deconditioning due to COVID-19.

## Introduction

As more people contract and recover from the Corona virus, knowledge of acute, post-acute and long-term physiological, physical, cognitive, and psychological sequelae evolve (Huang et al., 2021). Studies have reported people with COVID-19 who require hospitalization demonstrate long-term fatigue, cognitive difficulty, dyspnea, taste and smell impairments, muscle weakness, and poor cardiovascular endurance (Huang et al., 2021; Lopez-Leon et al., 2021; Wu et al., 2021; Zhang et al., 2021). Post-hospitalization, patients also commonly report anxiety, depression, post-traumatic stress disorder, and ICU-related neuropathy (Carenzo et al., 2021; Heesakkers et al., 2022). The majority of patients recovering from COVID-19 demonstrate impairments that hinder or restrict participation in activities of daily living (ADL), instrumental activities of daily living (IADL), the ability to live independently, return to work, and resume previous levels of social activity. Studies suggest early rehabilitation is associated with shorter recovery times and faster return to everyday activities (Choi et al., 2008; Coleman et al., 2017).

While studies recommend rehabilitation during the acute and post-acute phases of recovery (Demeco et al., 2020; Gutenbrunner et al., 2020; Sivan et al., 2020), little is known about the *depth* of rehabilitation needs because researchers have not utilized standardized assessment tools. Current studies examine patients 6–12 months post COVID-19 using screening tools too broad to provide detailed information about patients living in their home environment (Huang et al., 2021; Wu et al., 2021; Xiong et al., 2021; Zhang et al., 2021; Heesakkers et al., 2022). While longitudinal studies of sequelae offer critical information, a profile of the natural recovery during the early period after hospitalization is critical to improve recommendations for rehabilitation. The primary aim of this study was to identify post-hospitalization needs and services required for those diagnosed with COVID-19 using an interdisciplinary needs assessment with standardized rehabilitation tools. The secondary aim of this study was to report the natural course of recovery for people hospitalized with COVID-19 over a 30-day period using these standardized rehabilitation assessments.

## Materials and methods

We employed a modified, rehabilitation-oriented, rapid needs assessment using a longitudinal design to assess people who were discharged from hospital to home with a diagnosis of COVID-19 between April and December 2020. While a traditional needs assessment involves a reiterative process in which participants communicate needs to the researcher, in a *rapid needs assessment*, the timing for understanding health care needs is critical, thus the team begins with hypothetical, but informed areas of evaluation (Lee, 2019).

The team completed baseline measurements within 5°days of hospital discharge. We then assessed patients weekly over a 30-day period post-hospitalization using a battery of standardized tools utilizing nursing, occupational therapy, physical therapy, and social work utilizing cellular telephones, FaceTime, or Zoom platforms. Inclusion criteria included people at least 18 years of age, English speaking, diagnosed with COVID-19, hospitalized and subsequently discharged home, able to consent with or without caregiver assistance, and with internet access. Exclusion criteria included individuals who were discharged or met the criteria for hospice, demonstrated current drug or alcohol dependency, or who were pregnant. This study was approved by the Institutional Review Board of The University of Oklahoma Health Sciences Center (IRB#11988).

### Patient recruitment

During the course of this study, the IRB required research to be conducted virtually due to COVID-19 restrictions. We recruited participants from a convenience sample of patients admitted to a Level I Trauma Hospital on an academic health sciences center campus. We consented and provided participants a COVID Assessment Kit either personally prior to hospital discharge, or a combination phone call and front door drop-off. We utilized the “Evaluation for Consent” tool because people with COVID-19 are more likely to demonstrate cognitive impairment (Resnick et al., 2019; Sasannejad et al., 2019).

### Data collection

Advanced practice registered nursing staff (APRN), occupational therapists (OT)s, physical therapists (PT)s, and social work staff (SW) completed virtual interviews and physiological, physical, functional, cognitive, and mental health assessments. All study personnel utilized the secure Research Electronic Data Capture (REDCap) system to enter data.

Baseline assessments utilized:

### Evaluation to sign consent

Either APRN or SW staff determined each participant’s cognitive eligibility to consent using procedures described by Resnick et al. (2019).

### Sociodemographic information/medical history

Advanced practice registered nursing staff staff obtained sociodemographic, medical, and mental health history using the hospital chart and interview.

### Charlson co-morbidity index

This assessment characterizes patient comorbidities based on the International Classification of Function. Each co-morbidity has an associated weight from 1 to 6 based on the adjusted risk of mortality or resource use. The sum of all weights results in a single comorbidity score where “0” indicates no comorbidities. The higher the score, the more likely the predicted outcome will result in mortality or higher resource use (Charlson et al., 1987).

### Weekly standardized outcome tools

#### Physiological measures

##### Multi-dimensional dyspnea profile

The MDP assesses dyspnea intensity, sensory quality, unpleasantness, and affective distress using 12 items rated on a 0–10 numerical scale. The reliability, validity, and responsiveness to clinical change of the MDP in use for both acute and follow-up care is well-established (Meek et al., 2012; Banzett et al., 2015).

### Physical performance measures

#### SQUEGG hand strength test

The SQUEGG hand grip dynamometer measures grip strength up to 220°pounds using a smartphone application usable in the home environment. Traditional hand grip dynamometers have excellent reliability and validity (Mathiowetz, 2002).

#### Five times sit to stand test

The 5xSTS assesses strength, transitional movements, balance, and fall risk by documenting time required for a person to come to a complete stand from a sitting position five times. The 5xSTS has good reliability and validity (Schaubert and Bohannon, 2005; Bohannon, 2006; Tiedemann et al., 2008).

#### Timed up and go with manual and cognitive versions

The TUG comprises three separate tests to assess fall risk; under normal situations, with added physical stress (manual) and with divided attention (cognitive). Examiners assess the time it takes for a person to rise from a seated position, walk three meters, turn around, and return to sitting (normal), while carrying a glass 3/4 full of water (manual), and while counting backward by 3 or 4 from 100 (cognitive). TUG scores are predictive of fall risk with an 87% success rate, and have excellent reliability (Shumway-Cook et al., 2000; Hofheinz and Schusterschitz, 2010).

#### Borg rating of perceived exertion

The Borg RPE provides an estimate of heart rate during physical activity based on a rating scale ranging from 6 to 20 (Borg, 1982). Researchers have reported a high correlation between perceived exertion rating multiplied by 10, and the actual heart rate during physical activity (Borg, 1982; Marissa et al., 2008; Tabacof et al., 2022).

### Functional performance measures

#### Barthel index

The Barthel index uses an ordinal scale to measure and monitor change in activities of daily living (Table 3), with scores based on current ability (de Morton et al., 2008; Della Pietra et al., 2011). The Barthel index delivered by phone has excellent inter-relater reliability (Kappa = 0.90 with 985% CI, 0.85–0.94).

**TABLE 1:** Socio-demographic, patient chart data (*n* = 20), and data gathered by nursing staff over time reported at weeks 1 and 4 (mean values with 95% CI) (*n* = 15).

**Gender (*n* = 17, two missing–preferred not to answer)**	
Female	35.3%		
Male	64.7%		
**Age (*n* = 19)**			
0–39	31.6%		
40–49	21.1%		
50–59	26.3%		
60–69	15.8%		
80+	5.3%		
**Race/ethnicity (*n* = 18, one missing, preferred not to answer)**			
Caucasian	50.0%		
Hispanic (white)	2%		
Asian	11.1%		
Mixed	11.1%		
**BMI classification (*n* = 19)**			
Underweight	10.5%		
Normal weight	5.3%		
Overweight	15.8%		
Obese (I)	26.3%		
Obese (II)	10.5%		
Obese (III)	10.5%		
**Education (*n* = 19)**			
HS degree or GED	26.3%%		
Some college	21.1%		
College degree	21.1%		
Preferred not to answer	31.6%		
**Self-reported health status prior to COVID-19 (*n* = 15, four preferred not to answer)**			
Excellent	7.1%		
Very good	35.7%		
Good	21.4%		
Fair	35.7%		
**Chief complaint requiring hospitalization: (*n* = 19)**			
Fatigue	5.26%		
Fever	10.5%		
Shortness of breath	73.7%		
Other	10.5%		
**Number of days hospitalized (*n* = 19)**			
0–5 days	21.1%		
6–10 days	47.4%		
11–15 days	15.8%		
16–20 days	5.3%		
**Number of days requiring full ventilation (*n* = 19)**			
None	100%		
**Number of days requiring bi-level positive airway pressure (BiPAP) assistance with breathing (*n* = 19)**			
None	84.2%		
Eight days	5.3%		
Nine days	10.5%		
**Percentage of patients requiring supplemental oxygen at discharge (*n* = 16, missing three)**			
No	62.5%		
Yes	37.5%		
**Smoking status (*n* = 19)**			
Every day smoker	5.3%		
Some day smoker	10.5%		
Never smoked	47.4%		
Prefers not to answer	31.6%		
**Vaping status (*n* = 19)**			
Not currently using	100%		
**Self-reported previous diagnosis of anxiety or depression (*n* = 19)**			
Yes	7.1%		
No	21.4%		
Unsure	71.4%		
)**Self-reported previous thoughts of suicide (*n* = 19)**			
No	100%		
**Self-reported current thoughts of suicide (*n* = 19)**			
No	100%		
**Referral to physical or occupational services during hospitalization (*n* = 19)**			
Yes	15.8%		
No	84.2%		

**Nursing assessments**	**Week one**	**Week four**	*P-value* *

Multidimensional dyspnea profile MDP)	64.3 (44.3, 84.3)	13.2 (−7.2, 33.6)	**0.0059**
Weight	211.0 (177.7, 244.2)	205.0 (159.0, 250.9)	0.3750
Heart rate	94.9 (84.7, 105.0)	91.0 (79.9, 102.1)	0.01221
Systolic blood pressure (BP)	124.7 (114.4, 135.0)	127.6 (115.5, 139.6)	0.02563
Diastolic blood pressure (BP)	78.3 (72.3, 84.4)	79.8 (72.0, 87.5)	0.9302
Oxygen saturation levels	93.7 (92.4, 95.1)	94.2 (92.9, 95.4)	0.2773

*Wilcoxon signed rank test.

**TABLE 2:** Physical therapy data.

	Week one	Week two	Week three	Week four	*P-value* *
**Five times sit to stand (STS)**					
Five times sit to stand (sec)	17.5 (15.1, 19.9)	8.3 (6.6, 9.9)	13.1 (11.2, 14.9)	12.6 (10.9, 14.3)	**0.0009**
Borg for 5xSTS	10.7 (8.5, 12.8)	9.2 (7.2, 11.2)	7.8 (6.5, 9.2)	8.3 (6.0, 10.5)	**0.0146**
**Timed up and go (TUG)**					
TUG (sec)	15.1 (11.5, 18.6)	13.5 (9.9, 17.1)	11.6 (8.6, 14.7)	12.1 (8.9, 15.3)	**0.0419**
Borg for TUG	9.2 (8.0, 11.8	8.5 (6.7, 10.3)	7.5 (6.5, 8.4)	8.0 (5.9, 10.1)	0.1289
Manual TUG	14.3 (11.7, 16.9)	13.6 (10.5, 16.7)	12.3 (9.7, 15.0)	12.1 (9.4, 14.7)	0.0563
Borg for manual TUG	9.9 (8.0, 11.8)	8.6 (6.8, 10.5)	7.5 (6.6, 8.4)	8.3 (6.1, 10.6)	0.0508
Cognitive TUG	20.5 (13.8, 27.3)	17.1 (12.2, 22.1)	14.8 (10.8, 18.8)	13.7 (10.9, 16.6)	**0.0369**
Borg for cognitive TUG	9.4 (7.5, 11.4)	8.9 (6.8, 10.9)	7.8 (6.7, 9.0)	8.4 (6.2, 10.6)	0.0581
Squegg (hand grip strength)	65.0 (41.0, 88.9)	74.3 (50.5, 98.2)	77.3 (48.6, 106.0)	78.2 (56.5, 100.0)	**0.0020**

Mean values (with 95% CI) for the Five Times Sit to Stand test (5xSTS) in seconds, the Timed up and Go test (TUG) in seconds, and the Squegg hand grip strength test in pounds, and associated Borg rating of perceived exertion (RPE) using the 6–20 range scale during 4°weekly time points (*n* = 14). *P-value represents the difference in test values between week 1 and week 4, calculated by the Wilcoxon signed rank test. Bold values represent significance at the alpha = 0.05 level.

**TABLE 3:** Occupational therapy data.

	Week one	Week two	Week three	Week four	*P-value* *
**Barthel scale [maximum possible points for each category is in ()] For all Borg scale scores, 6 = minimal exertion**					
Feeding (10)	10.0**	10.0**	10.0**	10.0**	***
Borg for feeding	7.1 (5.7, 8.4)	6.5 (5.8, 7.2)	6.2 (5.9, 6.4)	6.3 (5.8, 6.8)	0.1250
Bathing (5)	4.0 (2.9, 5.1)	4.2 (2.9, 5.4)	4.6 (3.7, 5.5)	4.5 (3.4, 5.6)	0.5000
Borg for bathing	10.3 (7.9, 12.7)	8.8 (6.0, 11.5)	7.6 (5.6, 9.5)	7.8 (5.3, 10.3)	**0.0156**
Personal hygiene (grooming) (5)	4.7 (4.0, 5.4)	5.0**	4.6 (3.7, 5.5)	5.0**	1.0000
Borg for hygiene (grooming)	8.9 (7.0, 10.9)	7.5 (5.7, 9.3)	7.3 (5.2, 9.3)	7.2 (4.7, 9.7)	0.0625
Dressing (10)	9.0 (7.9, 10.1)	9.6 (8.7, 10.5)	9.6 (8.7, 10.5)	10.0**	0.25
Borg for dressing	8.9 (6.8, 10.9)	8.4 (6.5, 10.4)	8.2 (6.4, 10.0)	7.8 (5.8, 9.8)	**0.0313**
Bowel control (10)	10.0**	10.0**	10.0**	10.0**	***
Borg for bowel control	7.1 (5.6, 8.5)	6.3 (5.6, 7.1)	6.3 (5.6, 7.1)	6.7 (5.6, 7.8)	0.7500
Bladder control (10)	10.0**	10.0**	10.0**	10.0**	***
Borg for bladder control	6.5 (5.7, 7.2)	6.0**	6.0**	6.3 (5.6, 7.0)	1.0000
Toilet transfers (10)	10.0**	9.6 (8.7, 10.5)	9.6 (8.7, 10.5)	10.0**	***
Borg for toilet transfers	8.0 (6.4, 9.6)	7.3 (5.7, 8.8)	6.1 (5.9, 6.3)	6.1 (5.9, 6.3)	0.0625
Chair/bed transfers (20)	14.3 (13.4, 15.3)	15.0**	14.6 (13.7, 15.5)	15.0**	0.5000
Borg for chair/bed transfers	8.5 (6.8, 10.2)	7.8 (6.3, 9.2)	7.1 (6.1, 8.1)	6.3 (5.8, 6.8)	**0.0313**
Ambulation (15)	9.0 (5.7, 12.3)	9.6 (5.0, 14.2)	11.7 (8.0, 15.3)	13.0 (9.5, 16.5)	**0.0156**
Borg for ambulation	12.5 (9.7, 15.3)	10.4 (6.2, 14.7)	9.0 (5.7, 12.3)	9.2 (6.0, 12.5)	**0.0195**
Stair climbing (20)	5.4 (2.5, 8.2)	5.6 (2.0, 9.1)	5.6 (2.0, 9.1)	6.1 (2.4, 9.8)	0.2500
Borg for stair climbing	12 (8.4, 15.6)	10.7 (5.1, 16.4)	9.3 (3.7, 15.0)	10.0 (4.4, 15.6)	0.1563
**Lawton Instrumental Activities of Daily Living (IADL) scale (ranges from 0 = dependent through 2 = independent)**					
Ability to use the phone	2.0**	2.0**	2.0**	2.0**	***
Borg for using phone	7.1 (5.5, 8.8)	6.9 (4.9, 8.9)	7.3 (6.8, 5.1)	7.5 (4.9, 10.1)	***
Shopping	0.9 (0.4, 1.4)	1.3 (0.6, 1.9)	1.5 (1.0, 2.0)	1.4 (0.7, 2.1)	**0.0125**
Borg for shopping	11.0 (7.2, 14.8)	9.9 (6.5, 13.3)	8.3 (5.6, 11.0)	6.9 (5.7, 8.0)	0.0625
Food preparation	1.4 (0.9, 1.9)	1.4 (0.8, 1.9)	1.5 (1.1, 1.9)	1.8 (1.5, 2.1)	**0.0125**
Borg for food prep	7.6 (6.1, 9.2)	6.9 (5.4, 8.4)	7.5 (5.2, 9.7)	7.0 (5.0, 9.0)	0.3125
Housekeeping	1.0 (0.5, 1.5)	0.8 (0.2, 1.4)	1.5 (0.9, 2.0)	1.6 (1.0, 2.1)	**0.0313**
Borg for housekeeping	10.3 (7.6, 13.0)	6.8 (5.6, 8.1)	6.9 (5.8, 8.0)	6.8 (5.3, 8.2)	0.0625
Laundry	0.9 (0.4, 1.5)	1.1 (0.5, 1.7)	1.4 (0.8, 2.0)	1.8 (1.4, 2.1)	0.0625
Borg for laundry	9.6 (6.3, 12.9)	7.4 (6.0, 8.8)	7.9 (4.7, 11.1)	7.3 (4.6, 9.9)	0.0625
Mode of transportation	1.3 (0.8, 1.9)	1.3 (0.7, 2.0)	1.5 (0.9, 2.1)	1.8 (1.3, 2.3)	0.5000
Borg for transportation	8.1 (5.8, 10.4)	6.6 (5.1, 8.1)	6.7 (5.6, 7.7)	5.8 (5.8, 7.0)	0.0625
Responsibility for own medications	1.7 (1.4, 2.1)	1.8 (1.4, 2.1)	1.8 (1.4, 2.1)	1.9 (1.6, 2.1)	**
Borg for medications	6.4 (5.9, 6.8)	6.2 (5.9, 6.5)	6.2 (5.8, 6.6)	6.0 **	0.5000
Ability to handle finances	1.5 (1.1, 1.9)	1.7 (1.3, 2.1)	1.8 (1.6, 2.1)	2.0**	0.1250
Borg for finances	6.7 (6.0, 7.4)	6.1 (5.9, 6.3)	6.0**	6.0**	0.2500

**Five minute montreal cognitive assessment**	**Week One**		**Week Four**		***P-value***

Language (4)	2.7 (2.0, 3.4)		3.1 (2.5, 3.7)		0.5313
Orientation (6)	5.8 (5.5, 6.1)		6.0 **		1.0000
Memory (5)	3.4 (2.5, 4.3)		4.2 (3.3, 5.1)		0.4375
Total–5-Min MoCA (15)	11.7 (10.3, 13.1)		13.3 (12.1, 14.5)		0.1016

Mean values (with 95% CI) for the Barthel index for activities of daily living and the Lawton instrumental activities of daily living (IADL) scale in points [maximal points in ()], as well as the Borg RPE (rating of perceived exertion) using the 6 (minimal exertion)–20 (maximal exertion) range during these activities. *P-value represents the difference in test values between week 1 and week 4, calculated by the Wilcoxon signed rank test. **Scores across individuals are equal so no CI is available. ***Unable to compute because the mean difference between week 1 and week 4 ≈ 0. Bold values represent significance at the alpha = 0.05 level.

#### Lawton instrumental activities of daily living scale

The Lawton IADL Scale uses an interview format to assess independent living skills like phone use, shopping, food preparation, medications, finance, housekeeping, and laundry (Lawton and Brody, 1969). We modified scoring for more differentiation between participants using scores of 0 (dependent), 1 (partial assistance), and 2 (independent). The maximum score of 16 indicates self-reported independence. The tool demonstrates very high internal consistency and inter-rater reliability (Siriwardhana et al., 2018).

### Cognitive and psychological screening measures

#### Montreal cognitive assessment-5 minute protocol

The MoCA is a short cognitive screen predictive of mild cognitive impairment by assessing language, orientation, and memory using three items totaling a possible 15 points. The MoCA has good reliability and validity in differentiating cognitively impaired patients with executive domain impairment from those without and has excellent 30-day test-retest reliability (Pendlebury et al., 2013).

#### Patient health questionnaire-9

The PHQ-9 is a measure of depression using scores on nine items ranging from 0 (not occurring at all) to 3 (occurring nearly every day) for the “last 2°weeks” (Maurer, 2012). The PHQ-9 can be used to make a tentative diagnosis of depression in at-risk populations. When used as a screen for depression, the PHQ-9 has fair sensitivity and very good specificity (Maurer, 2012).

#### Generalized anxiety disorder-7

The GAD-7 is a measure of generalized anxiety with its potential causes using a seven-item scale with scores ranging from 0 (not occurring at all) to 3 (occurring nearly every day). Modeled after the PHQ9, it is quick (2–5 min) and effective when used within a primary health care setting, and can be self-administered or completed by interview, either electronically or in person (Roy-Byrne et al., 2009).

#### Procedures

The research team attended 8°h of study protocol training and received online written protocols for future reference. Training included strict study protocol adherence, standardizing assessments, assessing, and referral for patients experiencing medical deterioration, and documentation using the secure REDCap data collection system.

The COVID Home Care Kit contained an electronic scale, blood pressure cuff, mobile oxygen saturation monitor, SQUEEG hand strength dynamometer, and a 3-meter measuring tape. After receiving the kit, research personnel contacted participants to set up FaceTime, Zoom, and biomedical assessment tool technology. Personnel delivered the baseline assessments within 5°days of hospital discharge, and spread baseline assessments over 72 h to relieve patient and caregiver burden. Because anxiety is associated with COVID-19 (Heesakkers et al., 2022), our protocol included additional 5-min phone check-ins by nursing to assess physiological measures and recommend primary care physician follow-up if needed. Nursing staff tapered the frequency of these phone calls over 4°weeks calling 7°days during Week 1, 3°days during Week 2, 2°days during Week 3, and 1°day during Week 4. Disciplines communicated regularly about the time of scheduled visits to minimize risk of fatigue caused by multiple calls and assessments during the 30-day period. We asked participants at risk for falls, with significant ADL/IADL dependence, or with immediate health concerns to call his or her primary care physician for an appointment or referral for home health services.

#### Data analysis

Upon completion of the study, one researcher downloaded and analyzed all data using a combination of Microsoft Excel and SAS 9.4 (Carey, NJ, United States). Personnel computed summary statistics including means and 95% CI for all continuous variables, along with percentages for each categorical variable. To analyze change over the 4-week course of the study in each continuous variable, we utilized the Wilcoxon Signed Rank test with an alpha level equal to 0.05.

## Results

### Sample description

We enrolled the first 20 patients diagnosed with COVID-19 who consented upon discharge from a Level 1 Trauma Hospital on our academic campus. One patient dropped out immediately after enrolling, making our resulting sample size 19. Several patients failed to complete portions of the assessments, or did not participate after 1 or 2°weeks. Two thirds of participants self-identified as male and almost half were over 50 years of age. One third (35%) self-identified as Caucasian, with an additional 10% White-Hispanic. Although 85% had a BMI classification of overweight or obese (overweight = 16%, obesity type I = 32%, obesity type II = 26%, and obesity type III = 11%), two thirds responded their general health prior to COVID-19 was good, very good, or excellent (64%). Education ranged from high school or GED level through college graduate level. More than half of respondents reported living alone (Table 1).

Only 16% of our participants reported being every day or someday smokers and none used vaping devices. While 7% reported previous diagnoses of anxiety or depression, none reported thoughts of suicide, either currently, or in the past. The mean Charlson Comorbidity Index score was 3 out of a maximum score of 37, representing a low risk of either mortality or high levels of resource use. While nearly three quarters (74%) revealed their chief complaint requiring hospitalization was shortness of breath, no one in this cohort required full ventilation and only 16% required bi-level positive airway pressure (Bi-PAP) assistance with breathing. Almost half (47%) of the participants in this study were hospitalized 6–10 days. Only 16% of participants in the study received inpatient physical or occupational therapy, and none had a referral for outpatient or home health therapy services (Table 1).

### Physiological measures

The mean Week 1 Multidimensional Dyspnea Profile score was 64 (*M* = 64.3, 95% CI: 44.3–84.3), which dropped significantly to 13 (*M* = 13.2, 95% CI: −7.2 to −33.6) by week 4 (*p* = 0.0059). Heart rate also decreased significantly from 94.9 at Week 1 to 91.0 at Week 4 (*p* = 0.01). Other vital signs, including blood pressure, oxygen saturation levels, and weight remained stable over the 4°weeks following discharge (Table 1). Percentage of participants reporting fatigue dropped from 75% at Week 1, to 31% at Week 4 (*p* = 0.0031).

### Physical performance measures

The five times sit to stand test improved during the 4-week study from a mean 17.5 s to a mean 12.6 s (*p* = 0.0009). Participants did not fall below the cut-off time suggestive of further assessment for fall risk (12 s), during the course of the study. Timed Up and Go (TUG) scores improved during the 4-week study from a mean 15.1 s during Week 1, to a mean 12.1 s during Week 4 (*p* = 0.0419). Participants fell below the cut-off value suggestive of fall risk (13.5 s) after week two, meaning their fall risk was within an acceptable range. Both the Dual Task TUG (TUG-DT) and the Cognitive TUG (TUG-COG) also improved with mean values of 14.3 s and 20.5°s respectively during Week 1, to 12.1 s and 13.7 s during Week 4. These versions of the TUG represent a participant’s ability to engage cognitively or physically while executing complex motor tasks and acceptable fall risk levels are 14.5 and 15 s, respectively (Table 2).

BORG Perceived Rate of Exertion scores dropped dramatically during each of these physical exertion tests with highs of 10.7/20 during the 5xSTS test during Week 1 to 8.4/20 during the TUG-COG during Week 4. Final RPE scores for all these tasks fall within either the fairly light or very light ranges and are acceptable for physical tasks like those represented by the 5xSTS and the TUG (Table 2). Hand grip during the study changed from a mean 65 pounds in the dominant hand during week one, to a mean 78.2 pounds with the dominant hand during week four (*p* = 0.0020) (Table 2).

### Functional performance measures

Barthel ADL Index scores indicated all participants were independent in bowel continence, bladder continence, and toilet use upon discharge from the hospital. Barthel scores for bathing, dressing, hygiene (grooming), and transfers all approached or scored independence by the end of 4°weeks. Participants significantly improved in ambulation independence, beginning with a mean score of 9.0 points and ending with a mean score of 13.0 points (*p* = 0.0156). The ability to climb stairs was low at Week 1 with a mean of score of 5.4, and remained low at Week 4 with a mean score of 6.1 points. BORG Rating of Perceived Exertion scores demonstrated significant decreases in bathing, with a mean change from 10.3 to 7.8 points (*p* = 0.0156), in dressing with a mean change from 8.9 to 7.8 points (*p* = 0.0313), in bed and chair transfers with a mean change from 8.5 to 6.3 points (*p* = 0.0313), and in ambulation with a mean change from 12.5 to 9.2 points (*p* = 0.0195) Perceived exertion remained high for climbing stairs (Table 3).

Lawton IADL scores revealed participants were independent in their ability to use the phone at Week 1. In more physically and mentally complex tasks, while participants improved significantly in their ability to shop (mean change from 0.9 to 1.4, *p* = 0.0125), prepare food (mean change from 1.4 to 1.8, *p* = 0.0125), and do housekeeping (mean change from 1.0 to 1.6, *p* = 0.0313), scores did not indicate independence. While many Borg RPE scores for IADLs changed during the 4°weeks after discharge from the hospital, the changes were not significant (Table 3).

### Cognitive and psychological screening measures

The mean 5-min Montreal Cognitive Assessment test score in Week 1 was 11.7 points, indicating mild cognitive impairment. While this score improved to 13.3 points at Week 4, the difference was not significant (*p* = 0.10). Several participants demonstrated significant cognitive impairment that did not change or even declined during the course of the study (Table 3).

The mean GAD-7 total score during Week 1 was 5.9 points, which remained relatively consistent over the 4°weeks of the study ending with a mean during Week 4 of 4.5 points (*p* = 0.34). No individual variables of the GAD-7 changed significantly over time. The mean PHQ-9 score in Week 1 was 8.9 points, which reduced to a mean of 5.5 points in Week 4 (*p* = 0.10). No individual portions of the PHQ-9 changed significantly over time (Table 4).

**TABLE 4:** Social work data.

	Week one	Week four	*P-value* *
**Generalized Anxiety Disorder-7 (GAD-7), with scores ranging from 0 (not at all) to 3 (nearly every day) over the last 2°weeks**			
Feelings of nervousness	0.9 (0.3, 1.6)	0.9 (0.4, 1.5)	0.8125
Inability to stop worrying	0.9 (0.3, 1.5)	0.4 (0, 0.7)	0.3750
Excessive worry	0.7 (0.1, 1.3)	0.7 (0, 1.4)	1.0000
Restlessness	0.8 (0.2, 1.3)	0.5 (0.1, 1.0)	0.7500
Difficulty in relaxing	0.5 (0, 1.0)	0.2 (−0.1, 1.0)	0.3750
Easy irritation	1.2 (0.6, 1.8)	1.2 (0.5, 1.8)	1.0000
Fear something awful will happen	0.9 (0.3, 1.5)	0.5 (−0.2, 1.2)	0.1250
Total GAD-7 score	5.9 (2.4, 9.3)	4.5 (1.6, 7.4)	0.3438
**Patient Health Questionnaire–9 (PHQ-9) with values ranging from 0 (not at all) to 3 (nearly every day) over the last 2°weeks**			
Little interest or pleasure in doing things.	1.1 (0.5, 1.8)	0.6 (0.2, 1.1)	0.1250
Feeling down, depressed, or hopeless.	0.9 (0.2, 1.1)	0.4 (0.0, 0.7)	0.1563
Trouble falling or staying asleep, or sleeping too much.	1.4 (0.6, 2.1)	1.2 (0.2, 2.1)	0.5313
Feeling tired or having little energy.	1.9 (1.2, 2.7)	1.5 (0.6, 2.3)	0.4063
Poor appetite or overeating.	1.4 (0.7, 2.0)	0.6 (−0.1, 1.4)	0.3438
Feeling bad about yourself or that you are a failure or have let yourself or your family down.	0.4 (−0.1, 1.0)	0.2 (−0.1, 0.5)	0.5000
Trouble concentrating on things, such as reading the newspaper or watching television.	0.7 (0.2, 1.2)	0.6 (−0.1, 1.3)	0.4844
Moving or speaking so slowly that other people could have noticed. Or the opposite being so fidgety or restless that you have been moving around a lot more than usual.	1.1 (0.3, 1.8)	0.5 (0.1, 0.8)	0.2500
Thoughts that you would be better off dead, or of hurting yourself.	0.0**	0.0**	***
Total PHQ-9 score	8.9 (5.4, 12.3)	5.5 (2.3, 8.8)	0.0986

Mean values (with 95% CI) for the Generalized Anxiety Disorder-7 (GAD-7) and the Patient Health Questionnaire-9 (PHQ-9). *P-value represents the difference in test values between week 1 and week 4, calculated by the Wilcoxon signed rank test. **Scores across individuals are equal so no CI is available. ***Unable to compute because the mean difference between week 1 and week 4 = 0.

## Discussion

The primary aim of this study was to identify post-hospitalization needs and services that would allow patients diagnosed with COVID-19 to be as safe and independent as possible in their home settings using an interdisciplinary rapid needs assessment. In our sample of patients, discharge planning did not appear to include functional level or prognosis. Chart reviews revealed that 80% of our participants had not received any type of rehabilitation therapy and, when asked, were uncertain about how to progress their activity levels, or how to balance movement with rest. One partial explanation may be that training by professionals might have been poorly retained due to cognitive deficits, which were prevalent in week one. Further, we found significant impairments in physiologic, physical, functional, and cognitive performance which indicated the need for referral for a multi-disciplinary assessment and rehabilitation. These findings suggest a thorough assessment by nursing, occupational therapy, physical therapy, and social work staff could assist in clarifying post-discharge needs for patients transitioning to home after hospitalization for COVID-19.

The secondary aim of this study was to report the natural course of COVID-19 recovery over a 30-day period using standardized assessment tools. We found that while many measurements returned to normal or near normal over time, patients demonstrated increases in fall risk and loss of independence during their first few weeks at home, and required assistance with basic self-care. Caregivers were also impacted as they were unable to work unless they left impaired patients at home alone during initial recovery. Participants in our study did not demonstrate significant improvement in cognition over the 4-week period.

### Physiological measures

In this study, researchers monitored physiological measures of dyspnea, blood pressure, heart rate, and weight over 4°weeks. During week one, 75% of participants experienced dyspnea, compared to week four levels of 31%. This compares to a meta-analysis by Fernández-de-Las-Peñas et al. (2021), in which dyspnea decreased from a baseline level of 13.2%, to 27.2% at 60 days, and 26.3% at 90 days. Within our study, we found a significant decrease in heart rate, an insignificant decrease in weight and an insignificant increase in oxygen saturation levels. Patients with persistent dyspnea may benefit from referrals to professionals versed in respiratory and cardiac rehabilitation to improve their breath support and reduce their energy expenditure during functional activities.

Participants in this study were not highly impacted by comorbid conditions as evidenced by their mean Charlson Co-Morbidity Index score of three. Patients with comorbidities did experience poorer outcomes. Early identification of potential comorbidities during initial assessment, as well as enhanced attention to those potential complications during acute care, and discharge planning could assist in preventing secondary complications. Patients with comorbid conditions may also require enhanced time and rehabilitation hours compared to their counterparts without these conditions (Charlson et al., 1987; Choi et al., 2008).

### Physical performance measures

The participants in this study demonstrated significant levels of debilitation during their first week post hospitalization as evidenced by poor scores on the TUG, the 5x sit to stand, and the SQUEGG hand grip dynamometer. In previous studies, researchers have provided results on a 6-min Walk test. While none of our participants had the physical capacity to complete this test at hospital discharge, the 6-min walk test would have added a component of cardiovascular endurance to our measures, a factor we failed to adequately capture. By the end of week two, participants transitioned quickly to a safe level of walking and transfers and were no longer considered at fall risk. Although no participants reported falls in the 4°weeks after hospitalization, fall risk was high given their mobility status at discharge. While the physical performance assessments we utilized demonstrated improvement over the 30-day acute outpatient term, all three versions of the TUG along with the 5xSTS were probably unnecessary. Grip strength also increased significantly over 4°weeks, an important finding, as higher hand strength is associated with less mortality (Sayer and Kirkwood, 2015; De Biase et al., 2020).

### Functional performance

Participants in this study were independent in bowel and bladder control upon hospital discharge, however required assistance with all ADLs and basic mobility tasks until week four. Assessment scores suggested that patients continued to require assistance with bathing, ambulation, and stair climbing, even at week four. Participants with stalled performance scores also continued to have higher rates of perceived exertion.

Considering IADLs, our participants were independent with telephone use upon discharge although one reported shortness of breath while talking on the phone. While scores for financial and medication management quickly improved to normal, the OT assessment team reported these scores may have reflected ability versus observed performance, given the participant’s cognitive scores. Participants continued to be partially dependent in the IADL skills of shopping, food preparation, housekeeping, laundry, and transportation at week four. Most participants continued to need assistance due to mild shortness of breath and had Borg scores greater than seven. In support of our findings, Carenzo et al. (2021) reported 87% of the participants in their study were independent in self-care by 8°weeks post-hospitalization. Our findings suggest OT and PT referrals for patients with even mild ADL and IADL disability could minimize risk of secondary complications resulting from COVID-19 (He et al., 2015).

### Cognitive and psychological performance

Similar to other studies (Hampshire et al., 2021; Jaywant et al., 2021), our participant’s demonstrated mild cognitive impairment, particularly in the areas of language and memory. While these scores did not improve significantly over the 4-week trial they did trend upwardly. Participants continued to report problems with word-finding and short-term memory at week four and many requested information about how to enhance recovery. Hampshire et al. (2021) reported cognitive impairment and word-finding difficulty in their participants, and Jaywant et al. (2021) found impaired working memory in 55% of participants, impaired speed of processing in 40% of participants, and divided attention in 46% of participants recovering from COVID-19. Referrals to speech-language pathology and/or occupational therapy might enhance cognitive and communication ability (McGuire et al., 2006).

Unlike other studies (Xiong et al., 2021; Zhang et al., 2021), our participants did not experience significant or persistent self-reported anxiety and depression. Patients did report fears of re-infection, anxiety about financial concerns, and anxiety about not returning to baseline functional levels. The majorities of participants in our sample were married or had a caregiver staying with them. It is possible that social support moderated the level of anxiety noted in other studies (Viseu et al., 2018; Zhao et al., 2018). Clearly, mood should be monitored following COVID-19 as symptoms of depression and anxiety affect cognitive performance in older adults (Baune et al., 2006).

### Limitations

Because our samples of patients were never ventilator dependent, they most likely did not exhibit the most severe symptoms, therefore generalization to that population may be limited. Our sample size was relatively small, with some loss to follow-up. Our participants tired from meeting the demands of multiple phone calls on different days from multiple disciplines, suggesting a more streamlined approach may be beneficial. We were dependent on patient interpretation of test results as we did not conduct face-to-face assessments. We did ask caregivers to provide input when cognition may have impacted participant response reliability.

## Conclusion

We examined the post-discharge needs of patients hospitalized with COVID-19 and followed their natural recovery over 30 days without intervention. Our physiological, physical, cognitive, and functional findings suggest patients would benefit from assessment and intervention from a multi-disciplinary to address the range of deficits patients may experience as they recover from COVID-19. Early rehabilitation may shorten recovery time and allow patients to return to normal activities; foundational for an optimal quality of life.

## Data availability statement

The raw data supporting the conclusions of this article will be made available by the authors, without undue reservation.

## Ethics statement

The studies involving human participants were reviewed and approved by University of Oklahoma Health Sciences Center IRB. The patients/participants provided their written informed consent to participate in this study. Written informed consent was obtained from the individual(s) for the publication of any potentially identifiable images or data included in this article.

## Author contributions

CC contributed to the study conceptualization and design, developed the study manual and training, collected data and contributed significantly to the development of this manuscript. SJ contributed to the study design, led all team members in RED Cap training, collected and analyzed data, developed tables, and authored significant sections of the manuscript. HM and VL participated in data collection and manuscript development. AC-L, SD, WR, and TF participated in study conceptualization and design, trained team members, collected data, and edited all portions of the manuscript. All authors contributed to the article and approved the submitted version.

## References

[B1] Banzett R. B. O’Donnell C. R. Guilfoyle T. E. Parshall M. B. Schwartzstein R. M. Meek P. M. (2015). Multidimensional Dyspnea Profile: An instrument for clinical and laboratory research. *Eur. Respir. J.* 45 1681–1691. 10.1183/09031936.00038914 25792641 PMC4450151

[B2] Baune B. T. Suslow T. Engelien A. Arolt V. Berger K. (2006). The association between depressive mood and cognitive performance in an elderly general population–the MEMO study. *Dementia Geriatr. Cogn. Disord.* 22 142–149. 10.1159/000093745 16741362

[B3] Bohannon R. W. (2006). Reference values for the five-repetition sit-to-stand test: A descriptive meta-analysis of data from elders. *Percept. Mot. Skills* 103 215–222. 10.2466/pms.103.1.215-222 17037663

[B4] Borg G. A. (1982). Psychophysical bases of perceived exertion. *Med. Sci. Sports Exerc.* 14 377–381. 10.1249/00005768-198205000-00012 7154893

[B5] Carenzo L. Protti A. Dalla Corte F. Aceto R. Iapichino G. Milani A. (2021). Short-term health-related quality of life, physical function and psychological consequences of severe COVID-19. *Ann. Intensive Care* 11:91. 10.1186/s13613-021-00881-x 34089104 PMC8177269

[B6] Charlson M. E. Pompei P. Ales K. L. MacKenzie C. R. (1987). A new method of classifying prognostic comorbidity in longitudinal studies: Development and validation. *J. Chronic Dis.* 40 373–383. 10.1016/0021-9681(87)90171-8 3558716

[B7] Choi J. H. Jakob M. Stapf C. Marshall R. S. Hartmann A. Mast H. (2008). Multimodal early rehabilitation and predictors of outcome in survivors of severe traumatic brain injury. *J. Trauma Acute Care Surg.* 65 1028–1035. 10.1097/TA.0b013e31815eba9b 19001970

[B8] Coleman E. R. Moudgal R. Lang K. Hyacinth H. I. Awosika O. O. Kissela B. M. (2017). Early rehabilitation after stroke: A narrative review. *Curr. Atheroscler. Rep.* 19:59. 10.1007/s11883-017-0686-6 29116473 PMC5802378

[B9] De Biase S. Cook L. Skelton D. A. Witham M. Ten Hove R. (2020). The COVID-19 rehabilitation pandemic. *Age Ageing* 49 696–700. 10.1093/ageing/afaa118 32470131 PMC7314277

[B10] de Morton N. A. Keating J. L. Davidson M. (2008). Rasch analysis of the Barthel Index in the assessment of hospitalized older patients after admission for an acute medical condition. *Arch. Phys. Med. Rehabil.* 89 641–647. 10.1016/j.apmr.2007.10.021 18373993

[B11] Della Pietra G. L. Savio K. Oddone E. Reggiani M. Monaco F. Leone M. A. (2011). Validity and reliability of the Barthel index administered by telephone. *Stroke* 42 2077–2079. 10.1161/STROKEAHA.111.613521 21527755

[B12] Demeco A. Marotta N. Barletta M. Pino I. Marinaro C. Petraroli A. (2020). Rehabilitation of patients post-COVID-19 infection: A literature review. *J. Int. Med. Res.* 48 1–10. 10.1177/0300060520948382 32840156 PMC7450453

[B13] Fernández-de-Las-Peñas C. Palacios-Ceña D. Gómez-Mayordomo V. Florencio L. L. Cuadrado M. L. Plaza-Manzano G. (2021). Prevalence of post-COVID-19 symptoms in hospitalized and non-hospitalized COVID-19 survivors: A systematic review and meta-analysis. *Eur. J. Intern. Med.* 92 55–70. 10.1016/j.ejim.2021.06.009 34167876 PMC8206636

[B14] Gutenbrunner C. Stokes E. K. Dreinhöfer K. Monsbakken J. Clarke S. Côté P. (2020). Why Rehabilitation must have priority during and after the COVID-19-pandemic: A position statement of the Global Rehabilitation Alliance. *J. Rehabil. Med.* 50 317–325. 10.2340/16501977-2217 32719884

[B15] Hampshire A. Trender W. Chamberlain S. R. Jolly A. E. Grant J. E. Patrick F. (2021). Cognitive deficits in people who have recovered from COVID-19. *EClinicalMedicine* 39:101044. 10.1016/j.eclinm.2021.101044 34316551 PMC8298139

[B16] He S. Craig B. A. Xu H. Covinsky K. E. Stallard E. Thomas J. (2015). Unmet need for ADL assistance is associated with mortality among older adults with mild disability. *J. Gerontol. A Biol. Sci. Med. Sci.* 70 1128–1132. 10.1093/gerona/glv028 25834196 PMC4841172

[B17] Heesakkers H. van der Hoeven J. G. Corsten S. Janssen I. Ewalds E. Simons K. S. (2022). Clinical Outcomes Among Patients With 1-Year Survival Following Intensive Care Unit Treatment for COVID-19. *JAMA* 327 559–565. 10.1001/jama.2022.0040 35072716 PMC8787680

[B18] Hofheinz M. Schusterschitz C. (2010). Dual task interference in estimating the risk of falls and measuring change: A comparative, psychometric study of four measurements. *Clin. Rehabil.* 24 831–842. 10.1177/0269215510367993 20562166

[B19] Huang C. Huang L. Wang Y. Li X. Ren L. Gu X. (2021). 6-month consequences of COVID-19 in patients discharged from hospital: A cohort study. *Lancet* 397 220–232. 10.1016/S0140-6736(20)32656-8 33428867 PMC7833295

[B20] Jaywant A. Vanderlind W. M. Alexopoulos G. S. Fridman C. B. Perlis R. H. Gunning F. M. (2021). Frequency and profile of objective cognitive deficits in hospitalized patients recovering from COVID-19. *Neuropsychopharmacology* 46 2235–2240. 10.1038/s41386-021-00978-8 33589778 PMC7884062

[B21] Lawton M. P. Brody E. M. (1969). Assessment of older people: Self-maintaining and instrumental activities of daily living. *Gerontologist* 9 179–186. 10.1093/geront/9.3_Part_1.179 5349366

[B22] Lee J. (2019). Rapid needs assessment: An evidence-based model. *Eur. J. Train. Dev.* 43 61–75. 10.1108/EJTD-08-2018-0077

[B23] Lopez-Leon S. Wegman-Ostrosky T. Perelman C. Sepulveda R. Rebolledo P. A. Cuapio A. (2021). More than 50 long-term effects of COVID-19: A systematic review and meta-analysis. *Sci. Rep.* 11:16144. 10.1038/s41598-021-95565-8 34373540 PMC8352980

[B24] Marissa E. M. Denise M. C. Tom J. O. Robert J. P. (2008). Validity of values for metabolic equivalents of task during submaximal all-extremity exercise and reliability of exercise responses in frail older adults. *Phys. Therapy* 88, 747–756. 10.2522/ptj.20070161 18339798

[B25] Mathiowetz V. (2002). Comparison of Rolyan and Jamar dynamometers for measuring grip strength. *Occup. Ther. Int.* 9 201–209. 10.1002/oti.165 12374997

[B26] Maurer D. M. (2012). Screening for depression. *Am. Fam. Physician* 85 139–144. 22335214

[B27] McGuire L. C. Ford E. S. Ajani U. A. (2006). The impact of cognitive functioning on mortality and the development of functional disability in older adults with diabetes: The second longitudinal study on aging. *BMC Geriatr.* 6:8. 10.1186/1471-2318-6-8 16650284 PMC1472688

[B28] Meek P. M. Banzett R. Parsall M. B. Gracely R. H. Schwartzstein R. M. Lansing R. (2012). Reliability and validity of the multidimensional dyspnea profile. *Chest* 141 1546–1553. 10.1378/chest.11-1087 22267681 PMC3367480

[B29] Pendlebury S. T. Welch S. J. Cuthbertson F. C. Mariz J. Mehta Z. Rothwell P. M. (2013). Telephone assessment of cognition after transient ischemic attack and stroke: Modified telephone interview of cognitive status and telephone Montreal Cognitive Assessment versus face-to-face Montreal Cognitive Assessment and neuropsychological battery. *Stroke* 44 227–229. 10.1161/STROKEAHA.112.673384 23138443 PMC5593099

[B30] Resnick B. Gruber-Baldini A. L. Pretzer-Aboff I. Galik E. Buie V. C. Russ K. (2019). Reliability and validity of the evaluation to sign consent measure. *Gerontologist* 47 69–77. 10.1093/geront/47.1.69 17327542

[B31] Roy-Byrne P. Veitengruber J. P. Bystritsky A. Edlund M. J. Sullivan G. Craske M. G. (2009). Brief intervention for anxiety in primary care patients. *J. Am. Board Fam. Med.* 22 175–186. 10.3122/jabfm.2009.02.080078 19264941 PMC2896069

[B32] Sasannejad C. Ely E. Lahiri S. (2019). Long-term cognitive impairment after acute respiratory distress syndrome: A review of clinical impact and pathophysiological mechanisms. *Crit. Care* 23:1352. 10.1186/s13054-019-2626-z 31718695 PMC6852966

[B33] Sayer A. A. Kirkwood T. B. (2015). Grip strength and mortality: A biomarker of ageing? *Lancet* 386 226–227. 10.1016/S0140-6736(14)62349-7 25982159

[B34] Schaubert K. L. Bohannon R. W. (2005). Reliability and validity of three strength measures obtained from community-dwelling elderly persons. *J. Strength Cond. Res.* 19:717. 10.1519/R-15954.1 16095431

[B35] Shumway-Cook A. Brauer S. Woollacott M. (2000). Predicting the probability for falls in community-dwelling older adults using the Timed Up & Go Test. *Phys. Ther.* 80 896–903. 10.1093/ptj/80.9.896 10960937

[B36] Siriwardhana D. D. Hardoon S. Rait G. Weerasinghe M. C. Walters K. R. (2018). Prevalence of frailty and prefrailty among community-dwelling older adults in low-income and middle-income countries: A systematic review and meta-analysis. *BMJ Open* 8:e018195. 10.1136/bmjopen-2017-018195 29496895 PMC5855322

[B37] Sivan M. Halpin S. Hollingworth L. Snook N. Hickman K. Clifton I. J. (2020). Development of an integrated rehabilitation pathway for individuals recovering from COVID-19 in the community. *J. Rehabil. Med.* 52:jrm00089. 10.2340/16501977-2727 32830284

[B38] Tabacof L. Tosto-Mancuso J. Wood J. Cortes M. Kontorovich A. McCarthy D. (2022). Post-acute COVID-19 Syndrome Negatively Impacts Physical Function, Cognitive Function, Health-Related Quality of Life, and Participation. *Am. J. Phys. Med. Rehabil.* 101:48. 10.1097/PHM.0000000000001910 34686631 PMC8667685

[B39] Tiedemann A. Shimada H. Sherrington C. Murray S. Lord S. (2008). The comparative ability of eight functional mobility tests for predicting falls in community-dwelling older people. *Age Ageing* 37 430–435. 10.1093/ageing/afn100 18487264

[B40] Viseu J. Leal R. de Jesus S. N. Pinto P. Pechorro P. Greenglass E. (2018). Relationship between economic stress factors and stress, anxiety, and depression: Moderating role of social suort. *Psychiatry Res.* 268 102–107. 10.1016/j.psychres.2018.07.008 30015107

[B41] Wu L. Xiong H. Mei B. You T. (2021). Persistence of symptoms after discharge of patients hospitalized due to COVID-19. *Front. Med.* 8:761314. 10.3389/fmed.2021.761314 34881263 PMC8645792

[B42] Xiong Q. Xu M. Li J. Liu Y. Zhang J. Xu Y. (2021). Clinical sequelae of COVID-19 survivors in Wuhan, China: A single-centre longitudinal study. *Clin. Microbiol. Infect.* 27 89–95. 10.1016/j.cmi.2020.09.023 32979574 PMC7510771

[B43] Zhang X. Wang F. Shen Y. Zhang X. Cen Y. Wang B. (2021). Symptoms and health outcomes among survivors of COVID-19 infection 1 year after discharge from hospitals in Wuhan China. *JAMA Netw. Open* 4:e2127403. 10.1001/jamanetworkopen.2021.27403 34586367 PMC8482055

[B44] Zhao X. Zhang D. Wu M. Yang Y. Xie H. Li Y. (2018). Loneliness and depression symptoms among the elderly in nursing homes: A moderated mediation model of resilience and social suort. *Psychiatry Res.* 268 143–151. 10.1016/j.psychres.2018.07.011 30025285

